# Idiopathic Chronic Pancreatitis Presenting as Hemorrhagic Ascites: A Case Report

**DOI:** 10.7759/cureus.45303

**Published:** 2023-09-15

**Authors:** Noman Salih, Umair Amin, Khizer Hamza, Numan Ghani, Shahid Ali, Haider Sarfaraz, Muhammad Firdous Khan

**Affiliations:** 1 Internal Medicine, Hayatabad Medical Complex, Peshawar, PAK; 2 Pathology, Gajju Khan Medical College, Swabi, PAK; 3 Internal Medicine, Lady Reading Hospital, Peshawar, PAK; 4 Internal Medicine, Khyber Medical College, Peshawar, PAK; 5 Internal Medicine, Ayub Teaching Hospital, Abbottabad, PAK

**Keywords:** pancreatitis, hemorrhagic ascites, ileocecal junction, distention, upper abdominal pain, persistent, adolescent

## Abstract

This report presents a perplexing case involving a 16-year-old adolescent presenting with persistent upper abdominal pain and distention. The patient had no history of substance abuse or animal-related encounters. Clinical examination revealed abdominal tension, distention, and localized tenderness. Laboratory analysis indicated elevated white blood cell count, mildly reduced hemoglobin and platelet levels, and notably heightened amylase and lipase levels. Serum albumin displayed a minor decrease. Despite repeated consultations and ultrasound evaluations, the underlying cause remained elusive. Advanced imaging unveiled substantial abdominopelvic ascites, a shrunken pancreas with an expanded main duct, and thickening at the ileocecal junction. Ascitic fluid analysis unveiled hemorrhagic fluid with elevated cell and neutrophil counts. Notably, the fluid accumulation extended into the omental apron covering the intestines. Biopsy results ruled out malignancy and chronic infections. We diagnosed him as a case of idiopathic chronic pancreatitis presenting as hemorrhagic ascites. This case underscores the intricacies of diagnosing complex abdominal disorders. A comprehensive approach, involving multidisciplinary collaboration, rigorous diagnostic assessments, and meticulous patient evaluation, is essential for elucidating such challenging clinical scenarios.

## Introduction

Pancreatic ascites is a rare occurrence that arises from damage to the pancreatic duct, leading to continuous leakage of pancreatic fluids into the peritoneum [1]. The severity of this condition varies significantly and is often influenced by the location and extent of the ductal injury, as well as the presence of any fluid-related infections. While minor cases of pancreatic ascites typically resolve spontaneously, persistent instances coupled with an infection can give rise to substantial health complications, and in some cases, even death [1]. This notable case involves a perplexing medical scenario experienced by a 16-year-old boy - idiopathic hemorrhagic pancreatic ascites. This condition has garnered attention due to its uncommon nature. In the context of pancreatic disorders, the existence of sanguineous (bloody) fluid in the peritoneal and pleural spaces commonly suggests either hemorrhagic pancreatitis or pancreatic carcinoma accompanied by metastasis [2]. While the literature contains multiple reports of hemorrhagic ascites related to hemorrhagic pancreatitis, a comprehensive review of available literature has not revealed any documented instances of hemorrhagic ascites associated with chronic pancreatitis, except for a report detailing two cases of hemorrhagic ascites and hemothorax linked to benign pancreatic disease [2]. Mitchell CE documented a case of relapsing pancreatitis with recurrent pericardial and pleural effusions [3]. Gambill EE presented a case of acute hemorrhagic pancreatitis, analyzing a patient with disseminated fat necrosis, hypocalcemia, hypokalemia, uremia, diabetes mellitus, ascites, and bilateral hydrothorax [4]. Cases have also been documented concerning chronic pancreatic ascites and pancreatic pleural effusions, such as one reported by Cameron JL [5].

## Case presentation

We encountered a particularly intricate medical case involving a 16-year-old adolescent male who presented to our emergency department with persistent abdominal pain and swelling. The patient reported a history of enduring this abdominal discomfort for a span of seven to eight months. The pain was centered in the upper abdominal region and was characterized by a severity rating of 7 out of 10, along with mild tenderness. Additionally, the pain radiated to the back. Along with pain, he also complained of intermittent nausea and vomiting. There was no history of similar conditions in the siblings or any other relative.

Upon delving into the patient's medical history, we learned that he had sought medical attention from multiple local general practitioners in an attempt to alleviate the chronic abdominal pain. Regrettably, these previous medical visits had only resulted in the prescription of pain relievers and medications aimed at reducing gastric acid production. Despite undergoing several abdominal ultrasound examinations, the results consistently indicated normal findings. Importantly, the patient had no history of tobacco use, alcohol consumption, illicit drug utilization, or any incidents involving animals.

Upon the patient's initial presentation to our facility, we conducted a comprehensive assessment of his vital signs. His blood pressure measured 110/70 mmHg, pulse rate was 88 beats per minute, temperature was slightly elevated at 100.4°F, respiratory rate stood at 16 breaths per minute, and oxygen saturation was a healthy 98% on room air. During a thorough abdominal examination, we observed tension and distension in the patient's abdomen. Additionally, the umbilicus was centrally positioned and everted, while the flanks showed fullness. Mild tenderness was detected in the epigastric and umbilical regions. Notably, we also observed a positive fluid thrill during palpation. Chronic liver disease as a cause of ascites was ruled out based on the absence of anemia, jaundice, digital clubbing, or peripheral edema. Both the cardiovascular and respiratory assessments yielded unremarkable findings.

**Figure 1:**
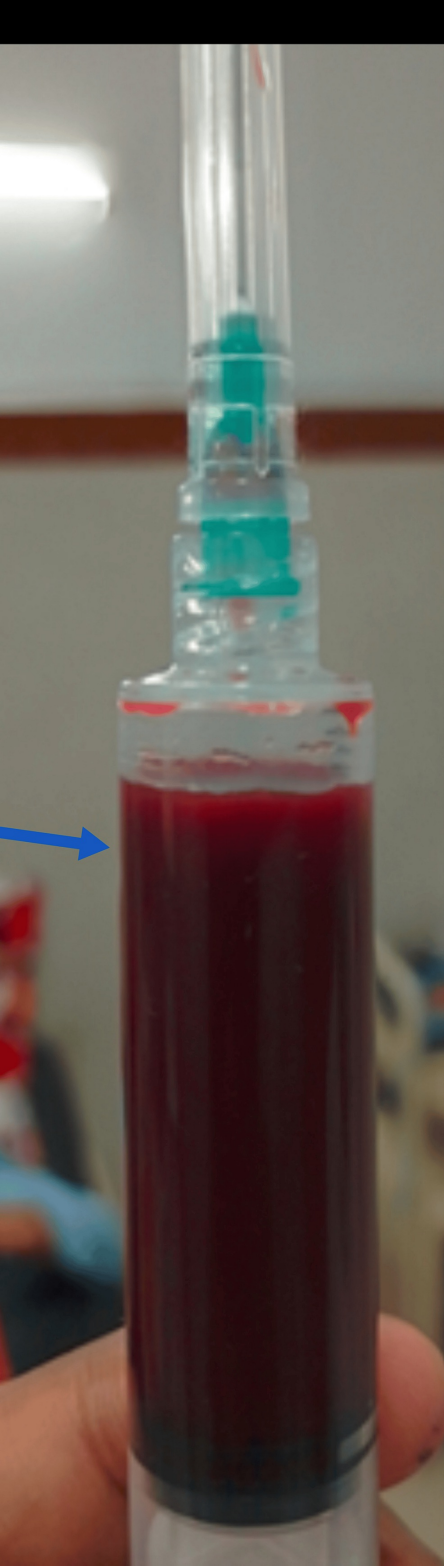
Blue arrow showing hemorrhagic ascites

The initial battery of laboratory investigations summarized in Table 1 provided noteworthy insights, including a white blood cell count of 19,000/μL (microliter), a hemoglobin level of 10.2 g/dL (slightly below the standard range), and a platelet count of 144,000/μL (microliter). The results of renal and hepatic function tests, as well as electrolyte levels, all fell within the expected range. A notable discovery was the elevated levels of serum amylase, a pancreatic enzyme involved in digestion, registering at 2297. Correspondingly, levels of lipase, another pancreatic enzyme, were elevated at 1019. Serum albumin levels measured 2.8, indicating a slight decrease, while the lipid profile remained within acceptable limits. Serum calcium levels were within the normal range. Importantly, tests for hepatitis B, hepatitis C, and human immunodeficiency virus (HIV) all returned negative results, confirmed through both rapid immunochromatographic test (ICT) and enzyme-linked immunosorbent assay (ELISA). Urinalysis revealed no anomalies. Coagulation studies demonstrated a prothrombin time/international normalized ratio (PT/INR) of 1.1 and an activated partial thromboplastin time (APTT) of 28 seconds, both within normal limits. Hemoglobin A1c (HbA1c) levels were within a healthy range, indicating glucose control over the past few months. Both the chest X-ray and echocardiogram (ECHO) of the heart exhibited normal findings. An ascitic tap was done and an examination of ascitic fluid revealed a hemorrhagic nature, as shown in Figure 1. The fluid had a high cell count (649), abundant red blood cells (4++++), an elevated presence of neutrophils (80%), and protein levels of 4.3. Notably, amylase levels in the ascitic fluid were significantly elevated at 3960. Calculation of the serum-ascites albumin gradient (SAAG) indicated a value of 0.8, suggesting local causes of the fluid buildup. Additional investigations included measuring cancer antigen 19-9 (CA 19-9, a tumor marker) and adenosine deaminase (ADA, an inflammation-associated enzyme) levels in the ascitic fluid, both of which were within normal ranges. Repeated checks for cancer cells in the ascitic fluid through cytology consistently yielded negative results.

**Table 1:** Laboratory investigations WBC: white blood cells; Hb: hemoglobin; PLT: platelets; PT: prothrombin time; APTT: activated partial thromboplastin time; INR: international normalized ratio; ALT: alkaline phosphatase; Na: sodium; K: potassium; Cl: chloride.

Test	Reference value	Absolute value
WBC (x10^3^/μL)	4000-11000	19,000
Hb (g/dl)	12.5-17.5	10.2
PLT (x10^3^/μL)	150000-400000	144,000
PT (sec)	12	14
APTT (sec)	28	28
INR	1	1.1
Total bilirubin (mg/dL)	0.1-1.0	0.6
ALT (U/L)	10-50	21
Alkaline phosphatase (U/L)	<390	94
Albumin (g/dL)	3.5-5.5	2.8
Amylase (U/L)	<90	2297
Lipase (U/L)	13-60	1019
Blood urea (mg/dL)	18-45	28
Creatinine	<90	0.582
Na (mmol/L)	135-145	140.4
K (mmol/L)	3.5-5.1	3.71
Cl (mmol/L)	96-112	98.3

**Figure 2:**
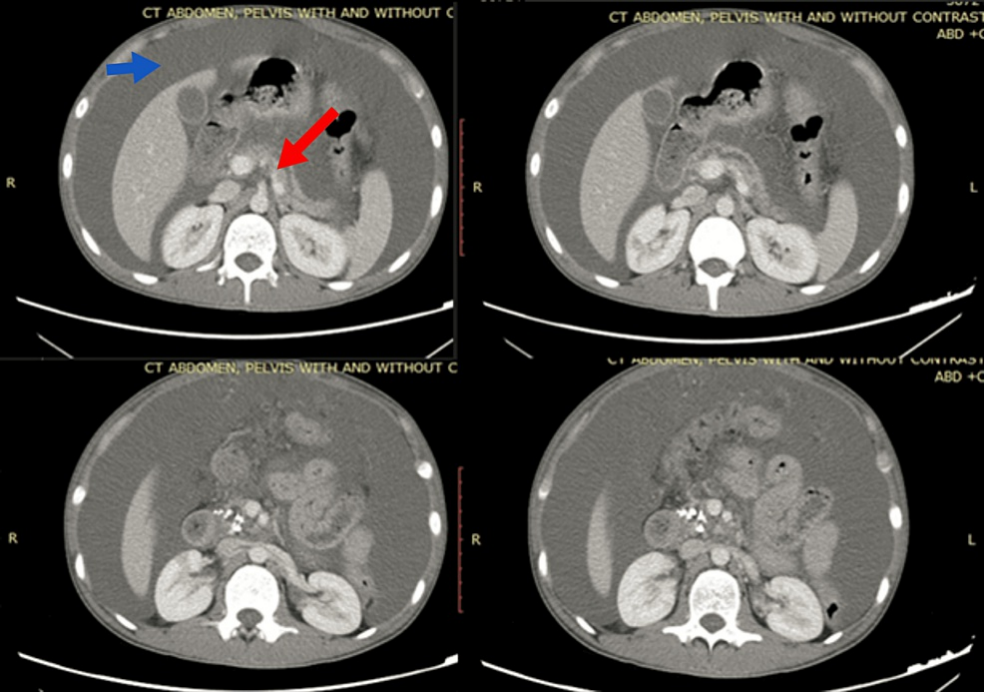
CT of the abdomen and pelvis with contrast The blue arrow showing ascites and the red arrow showing pancreatic calcification.

The radiological evaluation significantly enhanced our understanding of the case. The abdominal ultrasound revealed a substantial accumulation of fluid throughout the abdomen and pelvis. A subsequent contrast-enhanced computed tomography (CT) scan of the abdomen, depicted in Figure 2, provided valuable insights. It indicated a notably small pancreas with a dilated main pancreatic duct (MPD) and multiple small areas of calcium deposits in the pancreatic head. These findings strongly suggested chronic pancreatitis. Additionally, the CT scan revealed observable thickening at the ileocecal junction. Furthermore, the presence of abdominal ascites was accompanied by omental thickening and nodularity. These combined features prompted consideration for an omental biopsy.

**Figure 3:**
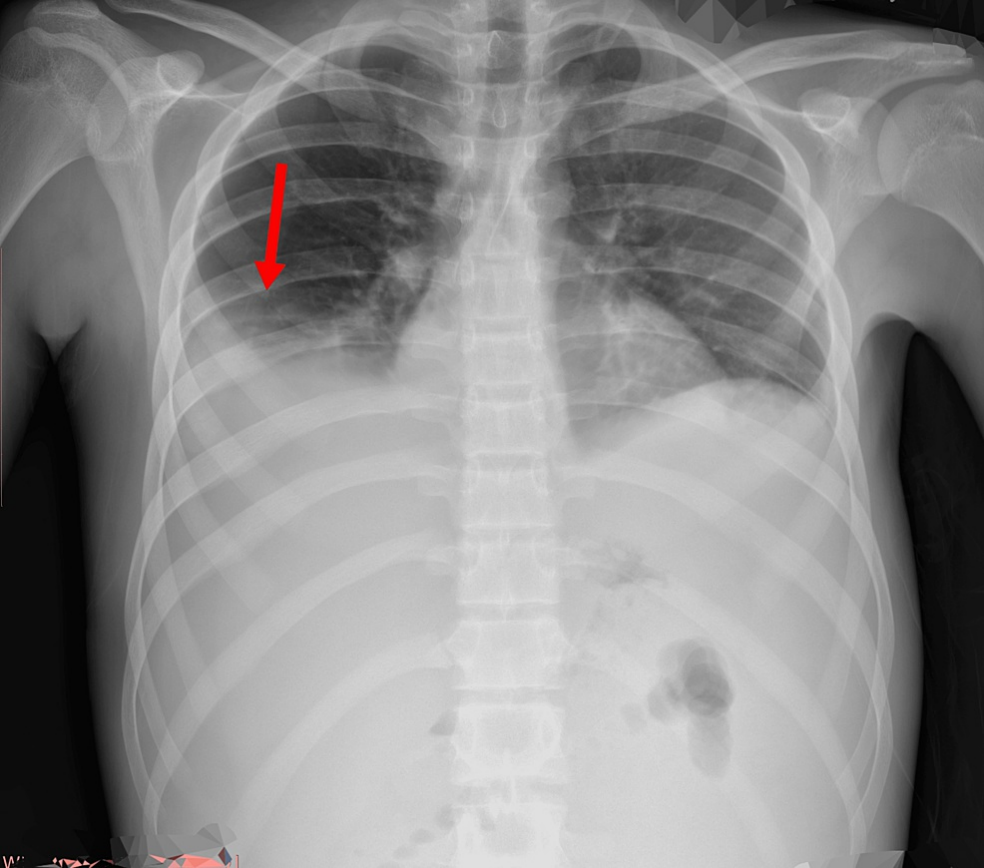
Chest X-ray Blunting of the right side pointed by a red arrow showing pleural effusions.

In response to the complex nature of the patient's presentation, we chose to perform a therapeutic ascitic tap. This procedure involves draining excess fluid from the abdomen to provide symptomatic relief. About a week later, the patient started experiencing shortness of breath. Physical examination revealed dull sounds upon percussion on both sides of the chest, indicating the presence of fluid. Furthermore, breath sounds were diminished, and vocal resonance was altered. A subsequent chest X-ray (Figure 3) unveiled fluid accumulation around the right lung. Upon tapping into this pleural fluid, we observed similar characteristics to the fluid found in the abdomen.

Guided by the insights gained from radiological assessments, we proceeded with an ultrasound-guided biopsy of the omentum, a fatty tissue apron that drapes over the intestines. The histopathological analysis of the biopsy specimen confirmed chronic pancreatitis and ruled out the presence of cancer and chronic infections such as tuberculosis, thereby contributing significantly to the diagnostic puzzle. His ascites and pleural effusions were drained and was put on symptomatic treatment and pancreatic enzyme replacement therapy. He underwent an extended hospital stay during which his condition gradually improved. Subsequently, he was discharged with a prescription for pancreatic enzyme replacement therapy. A follow-up appointment was scheduled after two weeks, during which his condition showed positive progress as the patient improved symptomatically. He received counseling on medication compliance and was advised to return if his condition deteriorated.

In summary, this intriguing case involving a 16-year-old patient highlights the complexity of diagnosing intricate abdominal conditions. A comprehensive approach, involving exhaustive testing, interdisciplinary collaboration among healthcare specialists, and meticulous radiological evaluations, played a pivotal role in deciphering the underlying factors contributing to the patient's complex presentation.

## Discussion

Let us delve into the intriguing case of the 16-year-old boy with a puzzling array of symptoms and findings that have left medical professionals scratching their heads. This complex scenario demands an in-depth discussion, highlighting the challenges in pinpointing a definitive diagnosis.

One possibility we are considering is chronic pancreatitis, a condition characterized by long-term inflammation of the pancreas. The CT scan results are consistent with this possibility, revealing a small pancreas, a dilated main pancreatic duct, and scattered calcium deposits. These findings correlate with the patient's history of persistent abdominal pain and the raised levels of pancreatic enzymes (amylase and lipase) in the blood. However, further tests like endoscopic retrograde cholangiopancreatography (ERCP) or magnetic resonance cholangiopancreatography (MRCP) might be required to confirm this diagnosis, providing a clearer picture of pancreatic function and any damage [6].

The presence of abdominal ascites (fluid accumulation in the abdominal cavity) paired with omental thickening and nodules detected through imaging raises a potential concern for secondary peritoneal malignancy. Although the initial ascitic fluid analysis did not reveal any malignant cells, it is worth mentioning that cytology has its limitations, and additional investigative measures such as an omental biopsy or exploratory laparoscopy might be necessary to rule this out [7].

The development of hemorrhagic pleural effusion (fluid around the lungs) adds another layer of complexity. The composition of the pleural fluid, rich in red blood cells and neutrophils, suggests a certain type of effusion. However, establishing a direct connection between hemorrhagic ascites and pleural effusion remains a challenge. The elevated levels of ascitic fluid amylase could potentially play a role, although further research is needed to establish a firm link [8].

While the patient's CA 19-9 levels are below the standard threshold for malignancy, they still warrant monitoring. This tumor marker can offer insights into gastrointestinal and pancreatic malignancies [9]. Also, given the high SAAG and the absence of clear cirrhosis indicators, we can likely rule out liver-related causes for his condition.

We should not disregard tuberculosis as a potential factor. Tuberculous peritonitis, characterized by abdominal pain, ascites, and even pleural effusion, could still be on the table. Despite the normal ascitic fluid ADA levels, we should exercise caution and explore this avenue further [10].

In essence, this case exemplifies the intricate nature of medical diagnosis. It is like fitting together a puzzle with numerous pieces, each representing a symptom, finding, or potential diagnosis. By combining clinical acumen, advanced imaging techniques, and collaboration among specialists, we are better equipped to decode this enigmatic case and offer the patient the most appropriate course of action.

## Conclusions

In conclusion, the case of the 16-year-old presenting with persistent abdominal pain and swelling vividly highlighted the intricacies of medical diagnosis. Despite enduring months of discomfort and previous medical consultations, a definitive diagnosis remained elusive. Through a meticulous process of examinations, laboratory tests, and advanced imaging, we gradually pieced together the puzzle.

The presence of elevated pancreatic enzymes, coupled with hemorrhagic ascitic fluid and significant radiological findings, strongly pointed toward chronic pancreatitis. The collaborative effort within our medical team played a pivotal role in guiding the treatment plan, which encompassed therapeutic procedures and enzyme replacement therapy. Despite the challenges posed by this intricate case, the patient's condition gradually improved over time. This journey underscored the paramount importance of comprehensive and persistent care in navigating complex medical scenarios such as this one.
